# Retrospective consent in critical care: how achievable is it?

**DOI:** 10.1186/2197-425X-3-S1-A138

**Published:** 2015-10-01

**Authors:** A Carins, D Shaw, A Wright, D Pearsons, A Strong, CH Toh, ID Welters

**Affiliations:** Royal Liverpool University Hospital, Intensive Care Unit, Liverpool, United Kingdom; Institute of Infection and Global Health, University of Liverpool, Liverpool, United Kingdom; Institute of Ageing and Chronic Disease, University of Liverpool, Liverpool, United Kingdom

## Introduction

Many critical care patients lack capacity due to disease or the need for sedation to facilitate invasive therapy. Research with incapacitated patients requires consent from a personal legal representative and upon regaining capacity retrospective consent from the patient [1]. Obtaining retrospective consent may delay data collection, requires additional research time, and consequently increases the cost of research.

## Objectives

Our aim was to assess the obstacles to obtaining informed consent for research in critical care. Outcomes included: the number of patients who gave full consent on recruitment, the number who gave retrospective consent, the number in whom death or discharge hindered retrospective consent, the time to retrospective consent and the number who withdrew assent given by the next of kin.

## Methods

Consent data was collected between 2008 and 2012 from patients enrolled in a prospective observational study of sepsis markers.1055 admissions to the Intensive Care Units of a tertiary teaching hospital were included. The inclusion criteria were: emergency admission and a stay of ≥ 48 hours. Planned admissions after elective surgery and repeat admissions were excluded. Specialist research nurses explained the design and purpose of the study, where possible full informed consent was obtained on enrolment. With incapacitated patients information was provided and assent was obtained from the next of kin with full consent sought from the patient when they regained capacity. Demographic data, APACHE II, length of ICU stay, outcome and diagnosis of sepsis were collected along with consent data.

## Results

985 patients met the inclusion criteria and 70 were excluded (53 repeat admissions, 11 elective admissions, three stay ≤ 48 h, one data error and three withdrawal of consent). The average age was 59 years and 43% of the patients were female. On enrolment 34% of patients gave full consent, 21% consented retrospectively, death or discharge prevented retrospective consent in 44%, 1% did not have or regain capacity and 0.3% withdrew consent after their next of kin had given assent. The average number of days between assent and retrospective consent was 27, with the longest duration being over five months and the shortest less than a day. 450 patients received two or more additional visits by research nurses in attempts to gain retrospective consent, with some patients receiving seven separate visits.

## Conclusions

Obtaining retrospective consent is time consuming, labour intensive and often unsuccessful. Very few patients for whom the next of kin gave assent later withheld consent. This brings into question the utility of retrospective consent. Further research is required to examine the relationship between attempts to obtain retrospective consent and withdrawal from clinical research.
